# The Rice Phosphate Transporter Protein OsPT8 Regulates Disease Resistance and Plant Growth

**DOI:** 10.1038/s41598-019-41718-9

**Published:** 2019-04-01

**Authors:** Zheng Dong, Wei Li, Jing Liu, Lihua Li, Sujun Pan, Saijun Liu, Jia Gao, Ling Liu, Xionglun Liu, Guo-Liang Wang, Liangying Dai

**Affiliations:** 1 Hunan Provincial Key Laboratory for Biology and Control of Plant Diseases and Insect Pests and College of Plant Protection, Hunan Agricultural University, Changsha, Hunan 410128 P. R. China; 2 Department of Plant Pathology, Ohio State University, Columbus, OH 43210 USA; 3 College of Agriculture, Hunan Agricultural University, Changsha, Hunan, 410128 P. R. China

## Abstract

The absorption of nutrients and disease resistance are two indispensable physiological processes in plants; however, it is still largely unknown whether there is cross-talk between their molecular signaling pathways. In this study, we identified the rice OsPT8 protein, which is a member of the phosphate transporters (PTs) Pht1 family and also plays a role in rice disease resistance. The transcriptional level of *OsPT8* is suppressed after infection with rice pathogens and treatment with pathogen-associated molecular patterns (PAMPs). Overexpression of *OsPT8* suppresses rice disease resistance against the pathogens *Magnaporthe oryzae* and *Xanthomonas oryzae* pv. *oryzae*. Accordingly, the transcription level of resistance related genes, such as *PAL* and *PBZ1*, is inhibited in plants overexpressing *OsPT8* (*OsPT8-*OX) after inoculation with these pathogens. In *OsPT8-OX* plants, PAMPs-triggered immunity (PTI) response genes, such as *OsRac1* and *SGT1*, are suppressed during treatment with PAMPs chitin or flg22. Moreover, the typical response of PTI is suppressed after chitin or flg22 treatment. We also identified OsPT8 as an interactor of a rice mitogen-activated protein kinase BWMK1, which is a regulator of disease resistance. Under low phosphate (Pi) conditions, the *OsPT8-*OX plants display better agronomic traits than the control plants. However, the differences in development between *OsPT8-*OX and the control plants are reduced upon the increase of Pi concentration. These results demonstrate that OsPT8 regulates the transduction of Pi signaling for development and negatively regulates rice immunity.

## Introduction

The growth and breeding of super rice varieties with low fertilizer demand, strong disease resistance, good quality, and high-yield are restricted by nutrient acquisition and defense response. Phosphorus (Pi) is one of the major macronutrients for the normal growth and development of almost all living species and plays a key role in plant metabolic processes; it is involved in vital substance synthesis, energy transfer, and signal transduction^1^. Plants absorb inorganic phosphate principally from soil. Therefore, the concentration of available Pi in the soil mainly limits plant growth and development, and Pi absorption remains a problem in agriculture^2^.

The absorption and transfer of Pi in plants rely on Pi transporters (PTs), which are classified into four subfamilies from Pht1 to Pht4^3^. The subcellular localization of the Pht1 subfamily is at the plasma membrane (PM), and there are 13 members (OsPT1-OsPT13) in the rice Pht1 subfamily^4,5^. For example, OsPT2 is a low-affinity PT that plays an important role in store Pi translocation in rice^6^. There is a Pi toxicity phenotype observed in transgenic rice overexpressing *OsPT2* by excessive uptake of Pi^7^. Altered expression of *OsPT4* affects the absorption and removal of Pi and also functions in the development of the embryo during the booting stage^8^. Contrary to OsPT2, OsPT8 is a high-affinity transporter for both Pi and arsenate, and *OsPT8*-OX plants tend to stockpile superfluous Pi involved in toxicity symptoms under high Pi conditions^9,10^.

Under Pi starvation conditions, plants adjust root architecture to maximize root superficial area by increasing the growth of lateral roots and root hairs to absorb Pi more effectively^11–13^. According to report, PTs and some Pi signal transduction related genes are involved in regulating root architecture based on Pi concentration^14–16^. For instance, compared with wild type plants, both the *OsPT1*-OX and *OsPT1*-RNAi plants can shorten root hairs under Pi-deficient conditions^14^. Similarly, the OsMYB2P-1 and OsARF16 also can regulate root architecture under Pi-starvation conditions, simultaneously influencing the expression level of PTs^15,16^.

The importance of phosphorus in the process of plant growth is unquestionable, but we still know little about the relationship between Pi absorb and disease resistance. An adequate supply of Pi is essential for crop growth and increases plant disease resistance^17^. In a previously described field trial, spraying 50 mM K_2_HPO_4_ increases rice yield by up to 32% and reduces neck blast caused by *Magnaporthe oryza*e (*M. oryzae*) by up to 42%^18^. However, the molecular mechanism of disease resistance enhanced by Pi treatment is still largely unknown. There are some genes that may function in both the Pi pathway and disease response such as AtPHT4;1 (also named ANTR1) which is a member of the *Arabidopsis* PHT4 family and localizes in leaf chloroplasts^19^. The dominant mutant of AtPHT4;1, *pht4;1-1*, is more susceptible to the virulent bacterium *Pseudomonas syringae* which is involved in SA-mediated plant defense^20^. Another study showed that AtPHT4;1 plays an important role in the defense response to pathogen infection and is regulated by a circadian clock protein CCA1^20,21^. In addition, the activity of ATP-synthase is inhibited in the *pht4;1-1* mutant, causing a dwarf phenotype with a lack of Pi^22^.

The mitogen-activated protein kinase (MAPK) cascade plays important roles in plant growth and development, hormone signaling, and immunity^23,24^. A MAPK cascade usually consists of a MAP kinase (MAPK), a MAPK kinase (MAPKK) and a MAPKK kinase (MAPKKK) at least. There are 15 MAPK genes have been reported in rice^25^. BWMK1 was the first reported rice MAPK, which is activated by a fungal elicitor and can enhance plant disease resistance against pathogens^26,27^. Mechanism analysis showed that BWMK1 enhances plant disease resistance depending on direct phosphorylating and activating the transcription factor OsEREBP1 in transgenic tobacco^26^. Another rice MAPK, OsMAPK6, can be activated by a sphingolipid elicitor, and regulated by the active form of a small GTPase OsRac1, which is involved in rice development and immunity^28^. A chitin-mediated elicitor OsMKK4 activates the OsMAPK3/OsMAPK6 to influence the plant metabolism of defense response and regulate the activation of OsWRKY53 during pathogen infection^29,30^. As a node, OsMAPK6 links a ternary complex formed by RAR1, SGT1 and Hsp90 to the OsRac1-mediated defense complex, and effects R gene-induced disease response and is involved in the accumulation of the reactive oxygen species (ROS)^28,31–33^.

To explore the possible functions of OsPT8 in cross-talk between plant defense responses and the Pi signaling pathway, we researched the underlying biological characteristics of OsPT8. In this study, we showed that OsPT8 regulates rice immunity, development and Pi transportation. Contrast with the wild type, the overexpression of *OsPT8* transgenic plants exhibit more susceptible to the rice fungal and bacterial pathogens. During the inoculation of pathogens and treatment of pathogen-associated molecular patterns (PAMP), the transcriptional levels of some disease resistance-related genes (such as *OsRac1*) were repressed compared with wild type. We also found that OsPT8 physically interacts with BWMK1. Under low Pi concentrations, *OsPT8*-OX plants show better agronomic traits compared with wild type. These results indicate that OsPT8 is involved in regulating the transduction of Pi signaling for plant development and plant immunity.

## Results

### OsPT8 is involved in plant immunity

**Figure 1:**
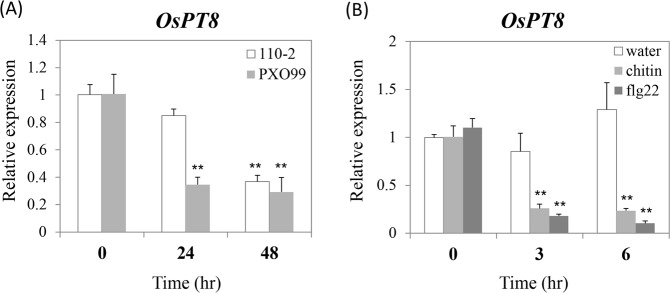
Relative gene transcription of the *OsPT8* in wild type (NPB) plants after rice pathogens inoculated or PAMPs treatment. (**A**) *OsPT8* relative transcription levels in NPB after *M*. *oryzae* isolate 110-2 or *Xoo* strain PXO99 inoculation; (**B**) *OsPT8* relative transcription levels in NPB after chitin or flg22 treatment. Error bars indicate the SD from three biological replicates (n = 3), Significance was determined level at **P < 0.01 (n = 3) with a t-test.

Although OsPT8 was reported that it functions on nutrient absorption and transport^9,10^, there remain unknowns about the signaling pathways of OsPT8. Therefore, to explore whether OsPT8 plays a role in rice disease resistance, we analyzed the *OsPT8* transcript level by quantitative RT-PCR in wild-type rice *Nipponbare* (NPB) after inoculation with the rice fungal and bacterial pathogens *M. oryzae* and *Xanthomonas oryzae* pv. *oryzae* (*Xoo*). Compared with normal expression of *OsPT8* at 0 hour post inoculation (hpi), inoculation with *M. oryzae* isolate 110-2 significantly suppresses the expression of *OsPT8* at 48 hpi (Fig. 1A). Similarly, the transcription of *OsPT8* was also inhibited after inoculation with *Xoo* strain PXO99 (Fig. 1A). Since PAMP-triggered immunity (PTI) is the main component of plant innate immunity, we examined whether *OsPT8* is involved in rice PTI. Punched the NPB leaf to disks and soaked them in the PAMPs chitin or flg22 solution to different time points (0, 3 and 6 h, respectively). Then, the results of the quantitative RT-PCR showed that the transcription level of *OsPT8* was down-regulated at 3 and 6 hour post treatment (hpt) after both chitin and flg22 treatment (Fig. 1B). These results suggest that *OsPT8* is involved in rice disease resistance and PTI.

### OsPT8 negatively regulates rice disease resistance

**Figure 2:**
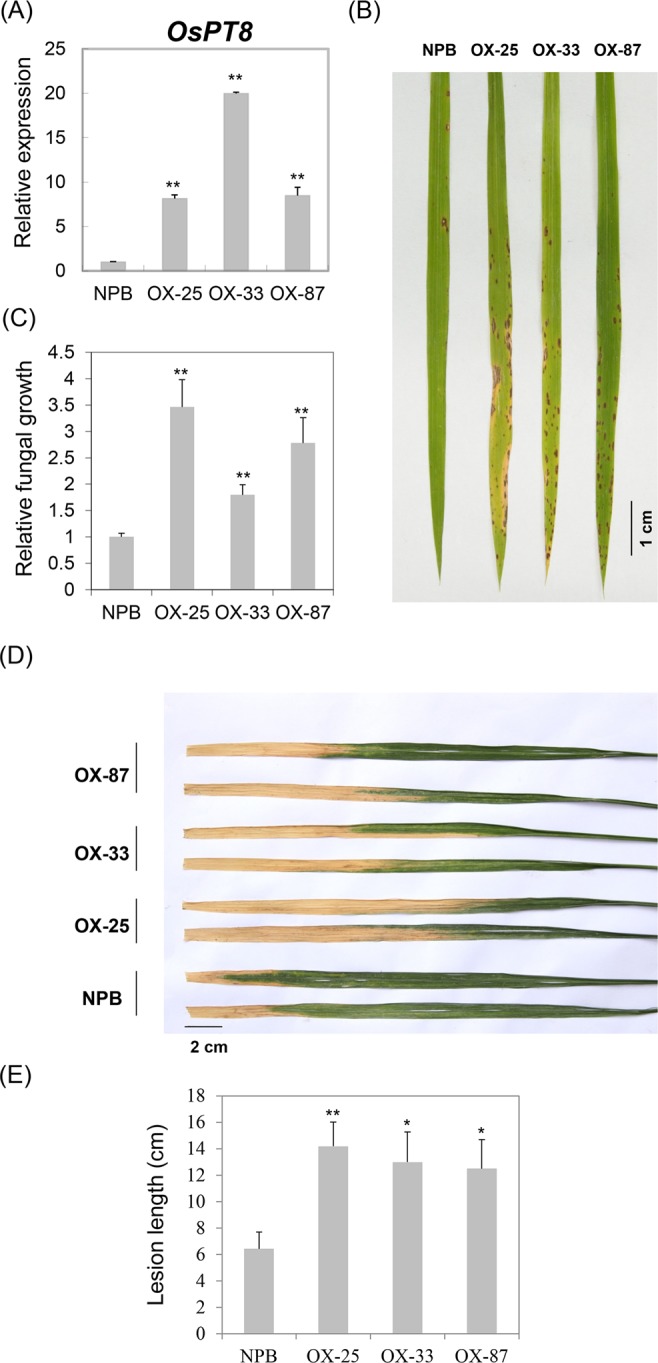
Disease phenotypes of the *OsPT8*-OX plants. (**A**) *OsPT8* relative transcription levels in *OsPT8*-OX plants. Error bars indicate the SD from three biological replicates (n = 3); (**B**) Disease phenotypes of the *OsPT8*-OX and wild-type (NPB) plants after spray inoculation with *M*. *oryzae* isolate 110-2 (Photographed at 7 DPI); (**C**) The relative fungal biomass of the *OsPT*8-OX and NPB plants after spray inoculation. Error bars indicate the SD from three biological replicates (n = 3); (**D**) Disease phenotypes of the *OsPT8*-OX and NPB plants after inoculation with *Xoo* strain PXO99 (Photographed at 14 DPI); (**E**) Bacterial blight lesion length in the *OsPT8*-OX and NPB plants. Error bars indicate the SD from three biological replicates (n > 15), Significance was determined level at *P < 0.05 and **P < 0.01 with a t-test.

To further understand the biological functions of OsPT8, we made *OsPT8* overexpression and RNAi transgenic plants with NPB background; the *OsPT8* overexpression and RNAi transgenic plants were challenged with rice fungal and bacterial pathogens, respectively. After inoculation with *M. oryzae* isolate 110-2, the *OsPT8* overexpressing plants showed severe disease symptoms with large lesions, while less disease lesions were observed in NPB plants at 7 days post infection (dpi) (Fig. 2A,B). Accordingly, the relative fungal biomass of the isolate in the inoculated leaves was significantly higher in the *OsPT8* overexpressing plants than in NPB plants (Fig. 2C). Similarly, the OsPT8 overexpressing plants were also more susceptible to *Xoo* strain PXO99 compared with NPB; the lesion length caused by PXO99 was much longer in *OsPT8* overexpressing plants than in NPB plants (Fig. 2D,E). However, the *OsPT8* RNAi plants have the same disease resistance level as NPB plants when they are inoculated with *M. oryzae* and *Xoo* (Fig. S1A–C). These results indicate that OsPT8 negatively regulates plant disease resistance against both fungal and bacterial pathogens.

**Figure 3:**
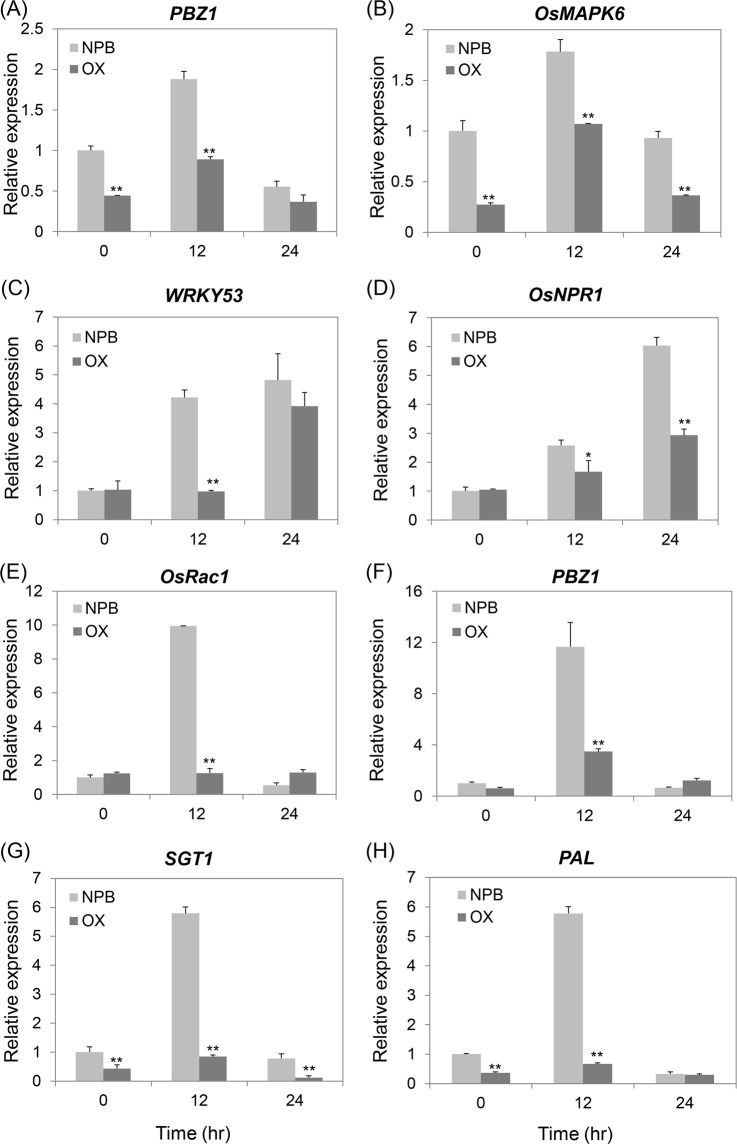
Defense-related genes transcription in the *OsPT8*-OX and wild type (NPB) plants after the rice blast pathogen *M*. *oryzae* or the bacterial blight pathogen *Xoo* inoculation. (**A**–**D**) Time-course relative transcripttion levels of *PBZ1*, *OsMAPK6*, *OsNPR1* and *WRKY53* in *OsPT8*-OX and NPB plants after *M*. *oryzae* isolate 110-2 inoculation. (**E**–**H**) Time-course relative transcription levels of *OsRac1*,* PBZ1*, *SGT1* and *PAL* in OsPT8-OX and NPB plants after *Xoo* strain PXO99 inoculation. Error bars indicate the SD from three biological replicates (n = 3), Significance was determined level at *P < 0.05 and **P < 0.01 (n = 3) with a t-test.

To determine the molecular pathway involving OsPT8 in the regulation of plant disease resistance, we compared the expression of pathogenesis related genes among *OsPT8* overexpression, *OsPT8* RNAi and NPB plants after inoculation with *M. oryzae* or *Xoo*. Under normal growth conditions, the transcript levels of the rice defense related genes *PBZ1* and *OsMAPK6* were suppressed in *OsPT8* overexpression plants compared with NPB (Fig. 3A,B). When infected with *M. oryzae*, the expression levels of the PR genes, such as *PBZ1*, *OsMAPK6*, *WRKY53* and *OsNPR1*, were obviously suppressed in *OsPT8* overexpressing plants compared with NPB at 12 or 24 hpi (Fig. 3A–D). In addition, the expression patterns of the defense related genes *OsRac1*, *PBZ1*, *SGT1* and *PAL* were analyzed in *OsPT8* overexpressing plants and NPB after inoculation with *Xoo*. These four genes in *OsPT8* overexpressing plants had much lower expression levels than those observed for NPB at 12 hpi (Fig. 3E–H). The transcript levels of the above defense-related genes showed no significant difference between *OsPT8* RNAi plants and NPB after *M. oryzae* and *Xoo* inoculation (Fig. S2A–D). These data indicate that *OsPT8* negatively regulates plant immunity through affecting pathogenesis-related genes.

### OsPT8 regulates PAMP-triggered immunity in rice

**Figure 4:**
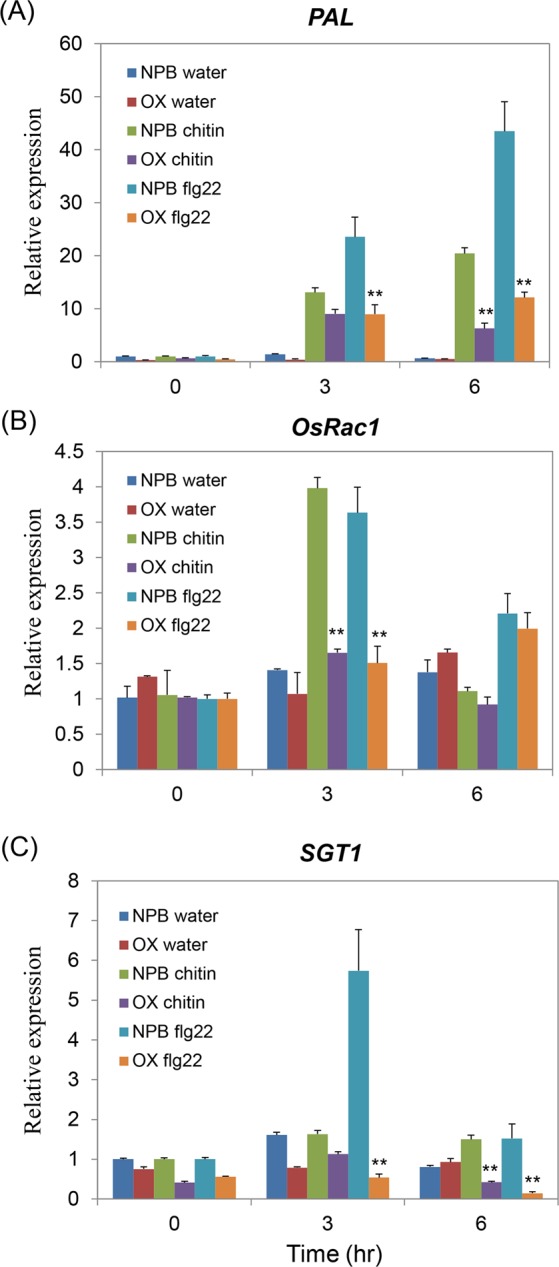
Defense-related genes transcription in the *OsPT8*-OX and wild type (NPB) plants after PAMPs chitin or flg22 treatment. (**A**–**C**) Time-course relative transcription levels of *PAL*, *OsRac1* and* SGT1* in *OsPT8*-OX and NPB plants after chitin or flg22 treatment. Error bars indicate the SD from three biological replicates (n = 3), Significance was determined level at *P < 0.05 and **P < 0.01 (n = 3) with a t-test.

To further analyze the function of OsPT8 on rice innate immunity, the *OsPT8* overexpression, *OsPT8* RNAi and NPB plants were treated with the PAMP elicitors (chitin and flg22) for PAMP-triggered immunity response detection. The quantitative RT-PCR revealed that the transcript level of the PTI pathway gene *PAL* was induced in both the *OsPT8* overexpression lines and NPB plants when treated with chitin or flg22, but the *PAL* transcription level was much lower in *OsPT8* overexpression plants compared with NPB (Fig. 4A). However, the expression of another PTI pathway gene, *OsRac1*, could not be induced by chitin or flg22 treatment in the *OsPT8* overexpression plants, but it could be activated by chitin and flg22 in NPB plants. This result indicates that *OsRac1* is also strongly suppressed in *OsPT8* overexpression plants during chitin and flg22 treatment (Fig. 4B). In addition, in *OsPT8* overexpressing plants the relative transcript level of the *SGT1* gene was only 1/10 of the transcription level in NPB after treatment with flg22 (Fig. 4C). We also detected the transcription of PTI-related genes in *OsPT8* RNAi plants after treatment with the two PAMP elicitors, and transcription of these genes was similar to the level determined in wide-type NPB, which showed a trend similar to the expression analyses observed in the investigations of inoculation and defense genes (Fig. S3A,B). These data indicate that *OsPT8* regulates both chitin and flg22-triggered rice immunity.

### OsPT8 interacts with BWMK1

**Figure 5:**
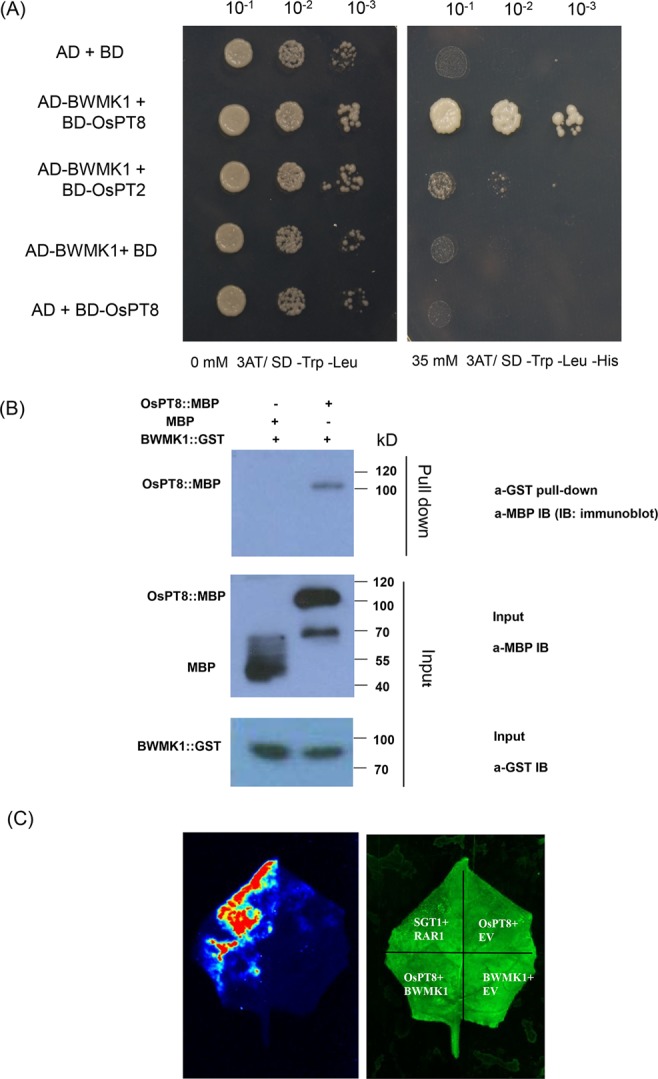
Interaction between OsPT8 and BWMK1. (**A**) OsPT8 interacts with BWMK1 in yeast. SD/-Leu-Trp-His medium containing 35 mM 3-amino-1,2,4-triazole (3′AT) was used to test the interaction; (**B**) The *in vitro* GST pull-down assay confirms the interaction between OsPT8: MBP and BWMK1: GST. MBP was used as a negative control. The amounts of proteins were examined by Western immunoblot analysis with anti-MBP or anti-GST antibody. These blots were exposed on different gels; (**C**) LCI assay show that OsPT8 interacts with BWMK1 in N. benthamiana leaves. SGT1/RAR1 was positive control.OsPT8/EV and BWMK1/EV were negative controls.

To further understand the functional mechanism of OsPT8 in plant defense responses, we aim to find out the interactor(s) of OsPT8. A system of yeast two hybrids (Y2H) was created to screen the interactional protein of OsPT8. We used OsPT8 as bait to screen the rice NPB cDNA library. Interestingly, we screened a MAPK protein BWMK1 interacting with OsPT8, and confirmed the interaction between BWMK1 and OsPT8 in the yeast cells (Fig. 5A). However, we did not observe an interaction between BWMK1 and OsPT2 which is homologue of OsPT8 in rice (Fig. 5A), suggested that the interaction between BWMK1 and OsPT8 in yeast is specific. In order to verify the reliability of this interaction in Y2H, a GST pull-down assay was applied to confirm the interaction between OsPT8 and BWMK1 (Fig. 5B). A firefly luciferase (LUC) complementation imaging (LCI) assay in *Nicotiana benthamiana* leaves was used to verify the interaction between OsPT8 and BWMK1. We observed strong LUC signal in the co-infiltration region of OsPT8-Cluc and BWMK1-Nluc vectors (Fig. 5C). The results showed that OsPT8 specifically interacts with BWMK1 both *in vitro* and *in vivo*.

### Overexpression of *OsPT8* in rice affects root and shoot growth

**Figure 6:**
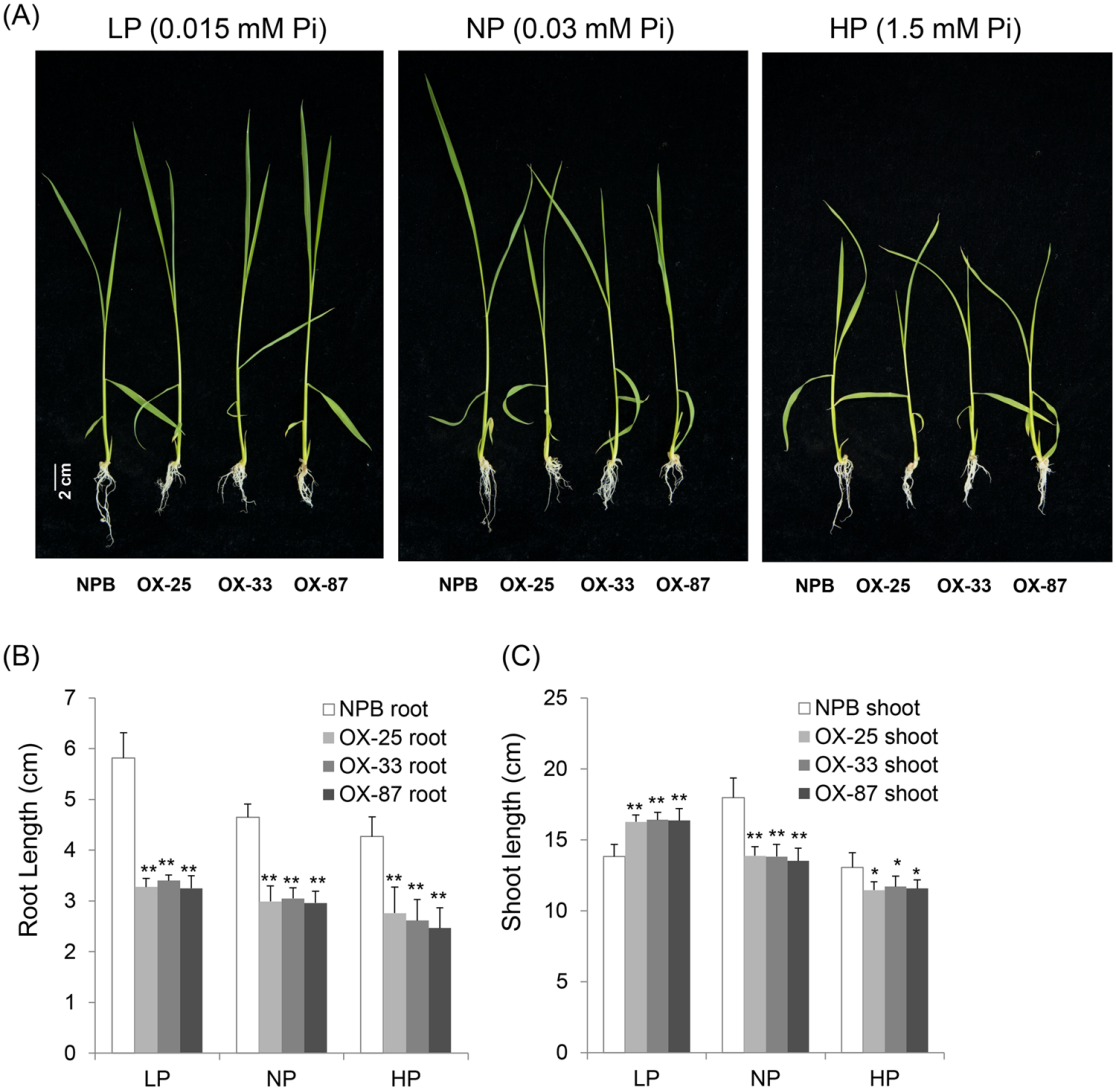
Growth phenotypes of the *OsPT8*-OX plants under different phosphate concentrations. (**A**) The phenotype of *OsPT8*-OX plants under different phosphate concentrations; (**B**), (**C**) The statistics data of root length and shoot length under different phosphate concentration. Error bars indicate the SD from three biological replicates (n = 9), Significance was determined level at *P < 0.05 and **P < 0.01 with a t-test.

Since OsPT8 is a Pi transporter in rice, we analyzed the Pi absorption function of OsPT8. Plants were treated with different Pi concentrations while grown on hydroponics to determine the effect of root and shoot growth. We found that OsPT8 affected rice root and shoot growth (Fig. 6A). The root length of *OsPT8*-OX plants was shorter than NPB under conditions with low (0.015 mM KH_2_PO_4_), normal (0.3 mM KH_2_PO_4_), and high (1.5 mM KH_2_PO_4_) Pi concentrations (Fig. 6A,B). This suggests that the overexpression of OsPT8 enhances the ability of rice to absorb Pi, which is not necessary to elongate root length and root surface to absorb enough Pi for the *OsPT8*-OX plants growth. On the contrary, the shoot length of *OsPT8*-OX plants was longer than the NPB under low Pi conditions, while shoot length of *OsPT8*-OX plants was shorter than NPB under normal Pi and high Pi conditions (Fig. 6C). Therefore, it is possible that the overexpression of *OsPT8* causes plants to absorb superfluous Pi under normal and high Pi conditions, which is toxic to plant growth, especially shoot growth. The *OsPT8*-RNAi plants show poor development under low Pi concentration condition, while once the Pi concentration is increased, the development of RNAi plants recovers to NPB level gradually (Fig. S4). Thus, we conclude that OsPT8 regulates plant root architecture through the Pi signaling pathway, and it also affects shoot development under different Pi concentrations.

## Discussion

### The function of OsPT8 in the regulation of rice innate immunity

BWMK1 is required for rice innate immunity^26,27,34^. Overexpression of *BWMK1* increases tobacco resistance against *Pseudomonas syringae* and *Phytophthora parasitica*^26^. BWMK1 phosphorylates a transcription factor of the AP2/ERF family, OsEREBP1, which enhances plant disease resistance to *Xoo* and confers drought tolerance in *OsEREBP1*-OX transgenic rice^26,27^. Similarly, we found that the phosphate transporter OsPT8, as the interactor of BWMK1, functions in plant disease resistance and Pi signaling. Our results showed that the transcription level of *OsPT8* decreases after infection with pathogens. Consistent with this finding, the line overexpressing *OsPT8* is more susceptible to fungal and bacterial rice pathogens, such as *M. oryzae* and *Xoo*, and genes related to disease resistance are suppressed in *OsPT8* overexpression plants before or after inoculation with pathogens compared with NPB. In addition, the PAMP-triggered immunity, a crucial component of plant innate immunity, is also significantly suppressed in *OsPT8*-overexpressing plants, and the transcription level of *OsPT8* is suppressed after chitin and flg22 treatment. However, unlike the *OsPT8* overexpression plants, there was no obvious difference between *OsPT8* RNAi plants and NPB in some defense responses. These results indicate that OsPT8 functions as a negative regulator of rice innate immunity.

In addition, we found that several rice immunity related genes (*OsRac1*, *OsMAPK6*, *SGT1* and *WRKY53*) in the OsRac1-mediated pathway were inhibited at the transcriptional level, indicating that OsPT8 directly or indirectly plays a role in this pathway. The interaction between OsPT8 and BWMK1 makes us realize that OsPT8 may be a point to link BWMK1 and OsRac1-mediated pathway. For the relationship between OsPT8 and BWMK1, we tend to propose a hypothesis that part of OsPT8 competitively binds to BWMK1 under the normal condition of plant growth. After pathogens infected, OsPT8 is down-regulated during pathogens infection, therefore, it releases BWMK1 to induce a series of downstream disease-related genes in the meantime. In the *OsPT8*-OX plants, the overmuch OsPT8 decreases the amounts of unbound BWMK1 and causes the suppression of the BWMK1-induced disease resistance. In the future, we need more research to explore the relationship between BWMK1 and OsRac1-mediated pathway to clarify the more detailed OsPT8-mediated plant disease resistance signaling network.

### OsPT8 is a regulator of rice growth and development

OsPT8 can reallocate Pi from vegetative organs to reproductive organs^35^. The Pi toxicity phenotype is observed in *OsPT8*-OX plants under 0.3 Mm Pi^9^. In addition to Pi absorption, the overexpression of *OsPT8* also facilitates the absorption of arsenate in rice and selenium in tobacco^10,36^. OsNLA1 is a RING-type E3 ubiquitin ligase that effects Pi homeostasis in rice by regulating the degradation of OsPT2 and OsPT8^37^. A previous study has reported that OsPT proteins are involved in rice root development^14^. In this study, we found that the *OsPT8*-OX plants have shorter shoots compared with NPB plants in normal phosphorus (NP) and high phosphorus (HP) conditions, suggesting that there is Pi toxicity in *OsPT8*-OX plants grown under NP and HP conditions. In contrast, under low phosphorus (LP) conditions, the shoot length of *OsPT8*-OX plants was longer than the root length in NPB, and with the decrease of the Pi concentration, the shoot length of *OsPT8*-OX plants gradually increased. Therefore, along with the decrease of over absorption of Pi, the suppression of shoot growth in *OsPT8*-OX plants is remedied. Unlike the shoot length, the root length of NPB was always greater than the root length of *OsPT8*-OX plants under HP, NP, or LP conditions. We speculate that because of the enhancement of *OsPT8*-OX plants to absorb Pi, the plant tends to decrease root surface area when it possesses sufficient ability of Pi absorption.

OsPT8 is also involved in both auxin- and Pi-mediated growth signaling pathways: when OsARF16, a cross-talk factor between auxin and Pi-starvation pathways, is knocked out, rice roots become insensitive to auxin and Pi treatment, and the expression level of *OsPT8* induced by Pi conditions is suppressed^16^. SGT1 is an OsRac1-mediated disease-resistance protein that reduced transcription in *OsPT8*-OX plants, which is also involved in the curling root phenotype by auxin-mediated pathways^38^. This study illuminated that OsPT8 functions as a cross talk regulator of plant innate immunity and plant growth signaling pathways.

## Materials and Methods

### Rice materials, growth, and transformation

The transgenic rice plants used in this study were developed from the Japonica cultivar *Oryza sativa Nipponbare* (NPB). After decortication, the rice seeds were germinated on 1/2 MS culture medium at 28 °C, 80% relative humidity (RH), and a 12 h light/12 h dark cycle.

pCAMBIA1301-*OsPT8*-OX and pFGC5941-*OsPT8*-RNAi were structured for *OsPT8* transgenic rice by *Agrobacterium*-mediated transformation.

### Hydroponic experiments with different Pi concentrations

One-week-old rice seedlings were transferred from 1/2 MS culture medium to IRRI nutrient solution containing 1.25 mM NH_4_NO_3_, 0.35 mM K_2_SO_4_, 1 mM CaCl_2_·2H_2_O, 1 mM MgSO_4_·7H_2_O, 0.5 mM Na_2_SiO_3_·9H_2_O, 20 mM Fe-EDTA, 20 mM H_3_BO_3_, 9 mM MnCl_2_·4H_2_O, 0.32 mM CuSO_4_·5H_2_O, 0.77 mM ZnSO_4_·7H_2_O, and 0.39 mM Na_2_MoO_4_·2H_2_O, at pH 5.5. Three different concentrations (LP, NP or HP) of KH_2_PO_4_ (0.015, 0.3, or 1.5 mM) as the only Pi source were individually added to separate solution to assess the resulting phenotypes. The plants in the hydroponic experiments were cultivated at 28 °C, 80% relative humidity, and a 12 h light/12 h dark cycle. The nutrient solution was adjusted to pH 5.5 every day and refreshed every 3 d.

### Inoculation with pathogens

To examine the resistance of plants to rice blast, 3-week-old rice seedlings were inoculated with *M. oryzae* isolate 110-2. The concentration of the spore suspension was 1.2 × 10^5^ spores/mL with 0.05% Tween-20. The infected plants were kept in the dark for 24 h before they were moved to greenhouse with a day cycle of 12 h light/12 h dark. Inoculated leaves were sheared and photographed at 7–9 dpi. The DNA of *M. oryzae* biomass was determined using quantitative PCR^39^. The *Xoo* strain PXO99 was used in a leaf-clipping method to infect the rice plants at the mature stage^40^. The 24 h dark treatment was essential before transferring the plants to a normal photoperiod. The lesion length was measured at 14 dpi.

### PAMPs treatment

Rice leaves were cut into pieces (1 cm^2^) and soaked in distilled water over night. Three pieces of each plant were placed into a 2.0 mL tube filled with 8 nM chitin or 100 nM flg22. The leaves were sampled at 0 h, 3 h and 6 h to analyze genes expression by quantitative RT-PCR.

### RNA isolation and quantitative RT-PCR

Total RNA was extracted from fresh rice tissue using Trizol reagent (Invitrogen). Appropriative RNA was treated with gDNA remover and cDNA synthesis kit (Trans one-step reverse transcription kit). Next, the cDNA was diluted for the 20 µL Trans qPCR system, then proceeded the real-time quantitative RT-PCR with a BioRAD CFX96 PCR amplifier. Three independent repeats were performed. The data were normalized with housekeeping gene ubiquitin.

### Yeast two-hybrid assay

The Quick & Easy yeast transformation mix system from TaKaRa was used in these assays. Full-length CDS of *OsPT8* and *BWMK1* was individually inserted into the AD vector (pPC86) or the BD vector (pDBLeu) separately. The yeast strain Mav203 was used for co-transformation and screened by synthetic dextrose (SD) medium without Leu and Trp (SD-Leu-Trp). For the interaction screening, 1 μL of each dilution (10, 100, 1000 times) was grown on SD-Leu-Trp-His medium with 0 mM or 35 mM 3-amino-1,2,4,-triazole (3′AT).

### GST pull-down

The full-length CDS of *OsPT8* or *BWMK1* gene was cloned into the pMAL-c4x or pGEX-6p-1 vector. Ten micrograms of OsPT8: MBP and 10 µg BWMK1: GST fusion protein was mixed together. And 60 µL of pre-rinsed glutathione sepharose beads (Promega) was co-incubated with the protein mixture for 4 h at 4 °C. The beads were then washed five times with 1 × TBST buffer. Finally, eluted protein from the beads and proceeded Western blot assay.

### LCI Assay

The full-length BWMK1 fragment was fused to the N-terminus of the pCAMBIA-NLuc vector, and full-length OsPT8 fragment also were fused to the C-terminus of the pCAMBIA-CLuc vector. These two vectors, NLuc/CLuc empty vectors and p19 were co-infiltrated into *N. benthamiana* leaves by *Agrobacterium* strain EHA105. The plants were kept at 26 °C (dark) for 48 h. The leaves were injected fluorescein potassium (cellgro) and photographed by Chemiluminescence imaging system (Bio-rad).

## Supplementary information

Supplementary information

## Data Availability

All data generated or analysed during this study are included in this published article.
