# Supplementary material for: The Rice Phosphate Transporter Protein OsPT8 Regulates Disease Resistance and Plant Growth
Source: Sci Rep. 2019 Apr 1;9:5408. doi: 10.1038/s41598-019-41718-9 (PMC6443681; doi:10.1038/s41598-019-41718-9)
Supplement: Supplementary file 1 — Supplementary information [file 41598_2019_41718_MOESM1_ESM.pdf]

The Rice Phosphate Transporter Protein OsPT8 Regulates Disease Resistance and Plant Growth

Zheng Dong[1,a], Wei Li[1,a,*], Jing Liu[1], Lihua Li[1], Sujun Pan[1], Saijun Liu[1], Jia Gao[1], Ling Liu[1], Xionglun Liu, Guo-Liang Wang[1,2], Liangying Dai [1,*]

[1] *Hunan Provincial Key Laboratory of Crop Germplasm Innovation and Utilization and College of Plant Protection, Hunan Agricultural University, Changsha, Hunan 410128, P.R. China.*

[2] *Department of Plant Pathology, Ohio State University, Columbus, OH 43210, USA*

[a] *These authors contributed equally to this work*

[*] *Correspondence: daily@hunau.net*; *liwei350551@163.com*

Supplementary information

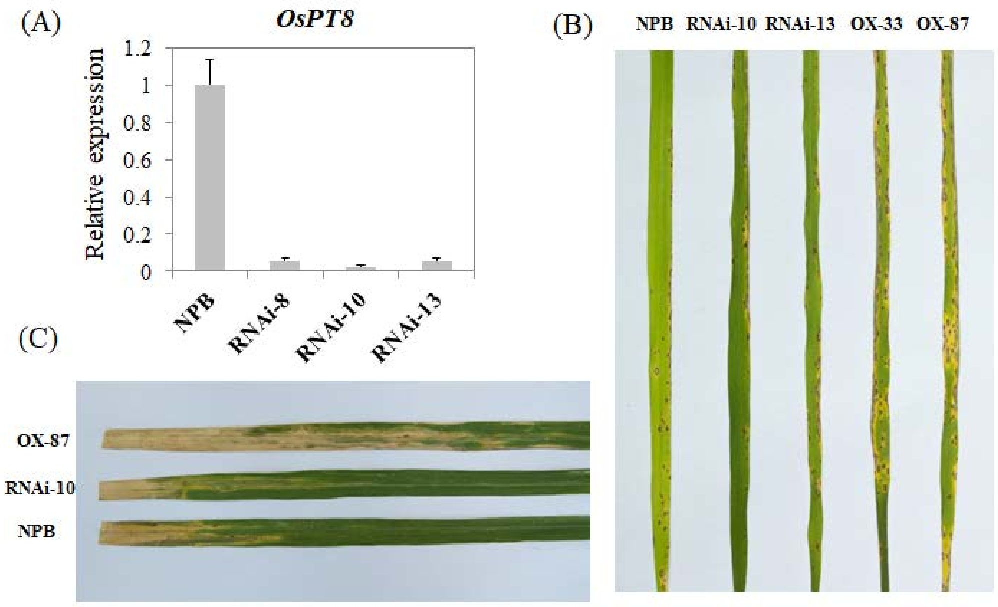

Figure S1. Disease resistance phenotypes of the *OsPT8* OX and RNAi plants. (A) *OsPT8* relative transcription levels in *OsPT8*-RNAi plants. Error bars indicate the SD from three biological replicates (n = 3); (B) Disease resistance phenotypes of the *OsPT8*-OX, *OsPT8*-RNAi and wild-type (NPB) plants after spray inoculation with *M. oryzae* isolate 110-2 (Photographed at 7 dpi); (C) Disease resistance phenotypes of the *OsPT8*-OX, *OsPT8*-RNAi and NPB plants after inoculation with *Xoo* strain PXO99 (Photographed at 14 dpi).

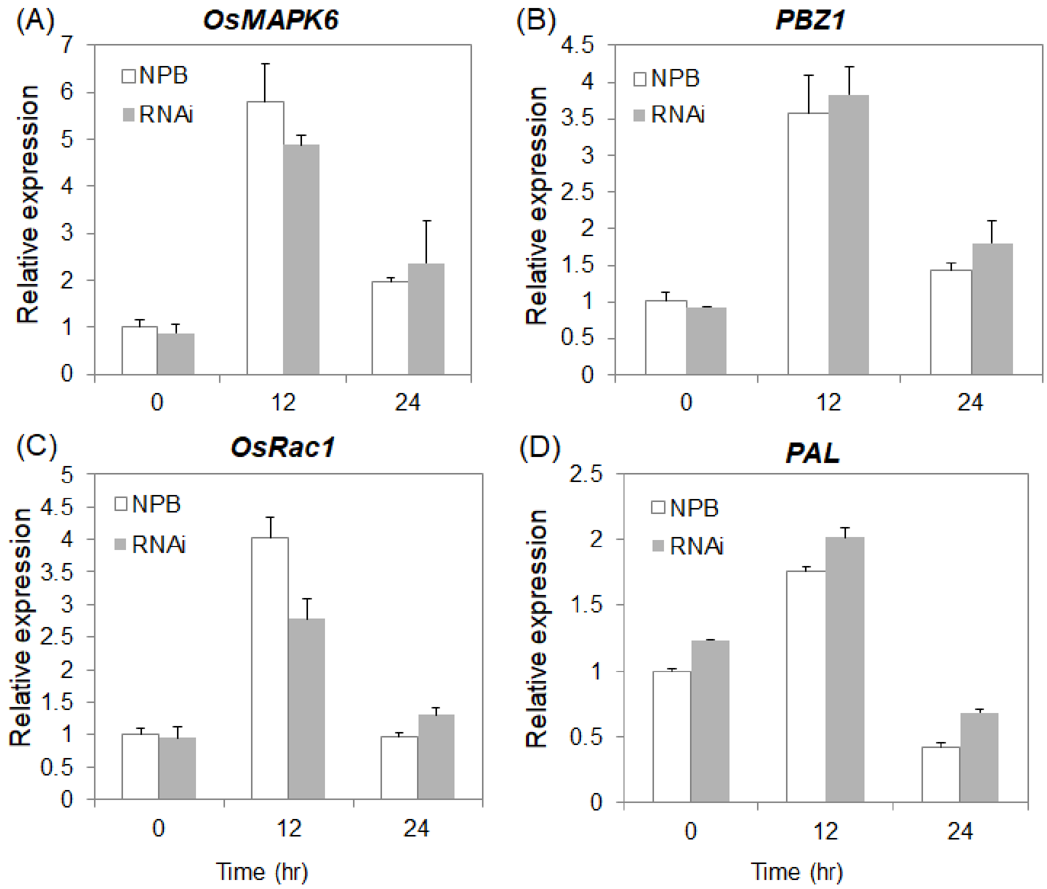

Figure S2. Defense-related genes transcription in the *OsPT8*-RNAi and wild type (NPB) plants after the rice blast pathogen *M. oryzae* or the bacterial blight pathogen *Xoo* inoculation. (A), (B) Time-course relative transcription levels of *MAPK6* and *PBZ1* in *OsPT8*-RNAi and NPB plants after *M. oryzae* isolate 110-2 inoculated; (C), (D) Time-course relative transcription levels of *OsRac1* and *PAL* in *OsPT8*-RNAi and NPB plants after *Xoo* strain PXO99 inoculation. Error bars indicate the SD from three biological replicates (n = 3).

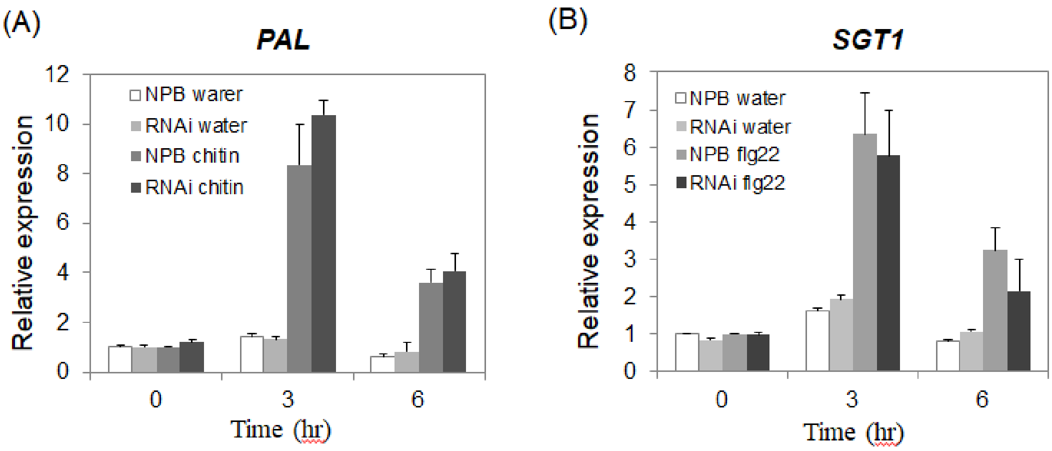

Figure S3. Defense-related gene expression of the *OsPT8*-RNAi and NPB plants after chitin or flg22 treatments. (A) Time-course relative transcript levels of *PAL* in *OsPT8*-RNAi and wild type (NPB) plants after chitin treatment; (B) Time-course relative transcript levels of *SGT1* in *OsPT8*-RNAi and NPB plants after flg22 treatment. Error bars indicate the SD from three biological replicates (n = 3).

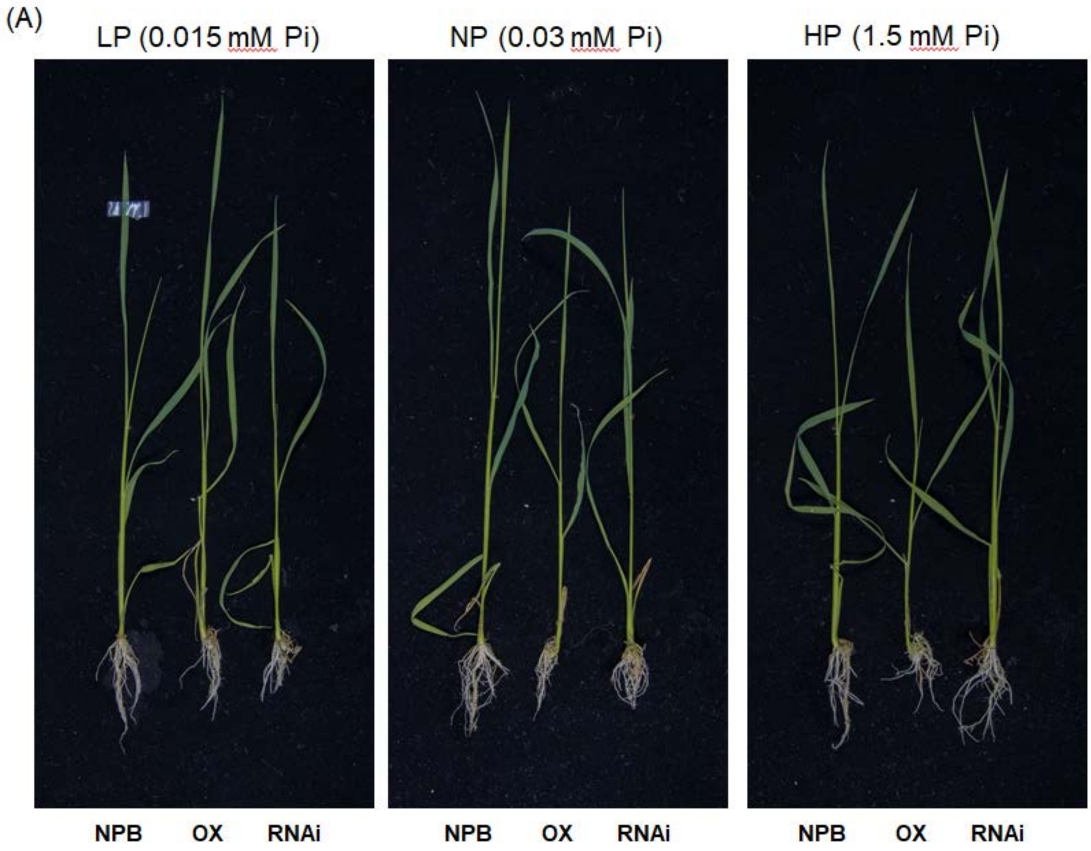

Figure S4. Growth phenotypes of the *OsPT8*-OX, *OsPT8*-RNAi and NPB plants under different phosphate concentrations.

Table S1. Primers used for qPCR

Name	ID	Sequence
OsPT8-QF	LOC4348741	5’-GCCAAGAACGCCAAGCAGGC-3’
OsPT8-QR		5’-GGAGAAGAGGCCGAAGCTGC-3’
PBZ1-QF	LOC4352491	5’-CCCTGCCGAATACGCCTAA-3’
PBZ1-QR		5’-CTCAAACGCCACGAGAATTTG-3’
OsRac1-QF	LOC4325879	5’-GCCCCATGGATCTACTCAGA-3’
OsRac1-QR		5’-AAAGGAGCTCCACTGCAGAA-3’
SGT1-QF	LOC4326682	5’-TCATCCCTGAGAAAAGCAGATAC-3’
SGT1-QR		5’-AAGGATATGATGGCCTCTGG-3’
PAL-QF	LOC4330040	5’-CTACAACAACGGGCTGACCT-3’
PAL-QR		5’-TCTGGACATGGTTGGTGATG-3’
OsMAPK6-QF	LOC4340170	5’-AGTCTGCTCATCACCCTTCAG-3’
OsMAPK6-QR		5’-ACCTTCAGCTTTGAACCAGGG-3’
OsNPR1-QF	LOC4327315	5’-TGAGAGTCTACGAGGAAGGTTGC-3’
OsNPR1-OR		5’-CGTTGTCTTTCAGGAGGTGGAT-3’
WRKY53-QF	LOC4338474	5’-AACACGGCAATACACACATAC-3’
WRKY53-QR		5’-TGACCTGTCTCTCAGCAGTTAC-3’
UBQ-QF	LOC4332169	5’-AACCAGCTGAGGCCCAAGA-3’
UBQ-QR		5’-ACGATTGATTTAACCAGTCCATGA-3’
MOPOT2-GQF	MGG_13294.6	5’-ACGACCCGTCTTTACTTATTTGG-3’
MOPOT2-GQR		5’-AAGTAGCGTTGGTTTTGTTGGAT-3’
OsUBQ-GQF		5’-TTCTGGTCCTTCCACTTTCAG-3’
OsUBQ-GQR		5’-ACGATTGATTTAACCAGTCCATGA-3’
